# Evaluation of Racial, Ethnic, and Socioeconomic Disparities in Initiation of Kidney Failure Treatment During the First 4 Months of the COVID-19 Pandemic

**DOI:** 10.1001/jamanetworkopen.2021.27369

**Published:** 2021-10-07

**Authors:** Kevin H. Nguyen, Rebecca Thorsness, Susan Hayes, Daeho Kim, Rajnish Mehrotra, Shailender Swaminathan, Navya Baranwal, Yoojin Lee, Maricruz Rivera-Hernandez, Amal N. Trivedi

**Affiliations:** 1 Department of Health Services, Policy, and Practice, Brown University School of Public Health, Providence, Rhode Island; 2 Department of Medicine, University of Washington School of Medicine, Seattle; 3 Sai University, Chennai, India; 4 Warren Alpert Medical School of Brown University, Providence, Rhode Island; 5 Providence VA Medical Center, Providence, Rhode Island

## Abstract

**Importance:**

Persons with kidney failure require treatment (ie, dialysis or transplantation) for survival. The burden of the COVID-19 pandemic and pandemic-related disruptions in care have disproportionately affected racial and ethnic minority and socially disadvantaged populations, raising the importance of understanding disparities in treatment initiation for kidney failure during the pandemic.

**Objective:**

To examine changes in the number and demographic characteristics of patients initiating treatment for incident kidney failure following the COVID-19 pandemic by race and ethnicity, county-level COVID-19 mortality rate, and neighborhood-level social disadvantage.

**Design, Setting, and Participants:**

This cross-sectional time-trend study used data from US patients who developed kidney failure between January 1, 2018, and June 30, 2020. Data were analyzed between January and July 2021.

**Exposures:**

COVID-19 pandemic.

**Main Outcomes and Measures:**

Number of patients initiating treatment for incident kidney failure and mean estimated glomerular filtration rate (eGFR) at treatment initiation.

**Results:**

The study population included 127 149 patients with incident kidney failure between January 1, 2018, and June 30, 2020 (mean [SD] age, 62.8 [15.3] years; 53 021 [41.7%] female, 32 932 [25.9%] non-Hispanic Black, and 19 835 [15.6%] Hispanic/Latino patients). Compared with the pre–COVID-19 period, in the first 4 months of the pandemic (ie, March 1 through June 30, 2020), there were significant decreases in the proportion of patients with incident kidney failure receiving preemptive transplantation (1805 [2.1%] pre–COVID-19 vs 551 [1.4%] during COVID-19; *P* < .001) and initiating hemodialysis treatment with an arteriovenous fistula (2430 [15.8%] pre–COVID-19 vs 914 [13.4%] during COVID-19; *P* < .001). The mean (SD) eGFR at initiation declined from 9.6 (5.0) mL/min/1.73 m^2^ to 9.5 (4.9) mL/min/1.73 m^2^ during the pandemic (*P* < .001). In stratified analyses by race/ethnicity, these declines were exclusively observed among non-Hispanic Black patients (mean [SD] eGFR: 8.4 [4.6] mL/min/1.73 m^2 ^pre–COVID-19 vs 8.1 [4.5] mL/min/1.73 m^2^ during COVID-19; *P* < .001). There were significant declines in eGFR at initiation for patients residing in counties in the highest quintile of COVID-19 mortality rates (9.5 [5.0] mL/min/1.73 m^2^ pre–COVID-19 vs 9.2 [5.0] mL/min/1.73 m^2^ during COVID-19; *P* < .001), but not for patients residing in other counties. The number of patients initiating treatment for incident kidney failure was approximately 30% lower than projected in April 2020.

**Conclusions and Relevance:**

In this cross-sectional study of US adults, the COVID-19 pandemic was associated with a substantially lower number of patients initiating treatment for incident kidney failure and treatment initiation at lower levels of kidney function during the first 4 months, particularly for Black patients and people living in counties with high COVID-19 mortality rates.

## Key Points

**Question:**

How did the volume and characteristics of patients initiating treatment for incident kidney failure change early in the COVID-19 pandemic, and were these changes associated with race, ethnicity, or socioeconomic status?

**Findings:**

In this cross-sectional study of 127 149 US adults with incident kidney failure during the first 4 months of the pandemic (March 1 through June 30, 2020), the number of patients initiating treatment for incident kidney failure declined by 30%, with Black patients and patients living in counties with high COVID-19 mortality initiating treatment with significantly worse levels of kidney function when compared with prior years.

**Meaning:**

These results suggest that declines in the number of patients with incident kidney failure may indicate delayed treatment initiation or changes in care delivery during the pandemic.

## Teaser

This cross-sectional study examines the volume of US adults initiating treatment for incident kidney failure across racial, ethnic, and socioeconomic groups during the first 4 months of the COVID-19 pandemic.

## Introduction

The coronavirus disease 2019 (COVID-19) pandemic has caused significant morbidity and mortality in the US, both directly and indirectly. The early months of the pandemic disrupted the provision of health care services unrelated to COVID-19 infection, with widespread reductions in hospitalizations and office visits for both discretionary and emergent services.^1,2,3^ Evidence suggests that many people did not receive necessary care because of shelter-in-place orders, fear of infection, limited availability, or loss of insurance coverage.^4^ The burden of the COVID-19 pandemic and these barriers to care have disproportionately affected racial and ethnic minority and socially disadvantaged populations, who are subject to structural inequities including racism, limited economic opportunity, and inequitable access to health care services.^5,6^ Moreover, delaying necessary care during the pandemic was more commonly reported by racial and ethnic minority populations and low-income families.^7^ These findings raise the urgency to understand the consequences of the pandemic on disparities in access to essential health services, in which delayed care can result in serious complications or death.

For people with kidney failure, kidney replacement therapy—including dialysis and kidney transplantation—is essential for survival. For patients with chronic kidney disease (CKD), missed routine care could result in faster progression to kidney failure, suboptimal treatment initiation, or urgent dialysis initiation during a hospitalization without vascular access in place, all of which are more common among racial and ethnic minority (particularly Black) patients.^8,9,10,11^ Additionally, the pandemic was accompanied by severe financial insecurity for low-income communities who disproportionately experienced loss of employment and health insurance coverage.^12,13^ This economic disruption exacerbated unmet social needs (eg, food insecurity or housing stability), which, among patients with CKD, are associated with more rapid declines in kidney function and faster progression to kidney failure.^14,15,16,17,18,19^

To date, there has been little information about how the pandemic factors into initiation of kidney failure treatment and on the distribution of demographic characteristics of patients with incident kidney failure initiating treatment. We examined changes in the numbers and characteristics of patients with incident kidney failure initiating treatment between January 2018 and June 2020. We stratified analyses by race and ethnicity, county-level COVID-19 mortality rate, and neighborhood socioeconomic disadvantage.

## Methods

### Study Design, Data Sources, and Study Population

We conducted a cross-sectional time-trend study of the changes in the number of patients with incident kidney failure initiating treatment and estimated glomerular filtration rate (eGFR), a measure of kidney function, from March 1 to June 30, 2020. The study population included all patients who developed and initiated treatment for incident kidney failure—hereafter referred to as patients with incident kidney failure—from January 1, 2018, to June 30, 2020, and who lived in the 50 US states or the District of Columbia. We used data from the Renal Management Information System Medical Evidence Form (CMS 2728), a national census of all patients with incident kidney failure initiating long-term dialysis or receiving a preemptive kidney transplantation.^20^ The CMS 2728 form is completed irrespective of insurance coverage, treatment modality, or citizenship, and includes detailed sociodemographic characteristics, clinical information, and patients’ primary mailing address. We geolocated incident patients to US Census block-groups using ArcGIS spatial mapping software version 10.5.1 (ESRI). Brown University’s institutional review board and the Centers for Medicare & Medicaid Services (CMS) Privacy Board approved the study protocol and waived the need for informed consent owing to use of deidentified data. This study follows the Strengthening the Reporting of Observational Studies in Epidemiology (STROBE) reporting guideline for cross-sectional studies.

To determine neighborhood socioeconomic disadvantage, we linked the CMS 2728 data to the 2018 Area Deprivation Index (ADI) at the census block-group level. The ADI is an area-level measure of disadvantage based on 17 metrics of income, education, employment, and housing quality.^21,22^ To identify whether a patient lived in a county with high COVID-19 mortality, we used daily, county-level cumulative COVID-19 deaths and publicly available 2020 US Census Bureau annual county population estimates.^23,24^

### Measures

The primary outcomes were: (1) the monthly number of patients initiating treatment for kidney failure, which included both incident dialysis patients and those receiving preemptive kidney transplantation; and (2) eGFR at the time of treatment initiation. We calculated eGFR using serum creatinine values reported on the CMS 2728 using a modified Chronic Kidney Disease Epidemiology Collaboration formula excluding the race term.^25,26,27,28^ We estimated the difference between observed and expected kidney failure incidence based on historical trends to capture changes in incidence associated with the COVID-19 pandemic.

We also assessed changes in sociodemographic and clinical characteristics of patients with incident kidney failure. Sociodemographic characteristics included age, sex, race and ethnicity, health insurance coverage, and employment status. Race and ethnicity information was collected by the dialysis provider or transplantation facility based on patient self-report at treatment initiation and classified as non-Hispanic White, non-Hispanic Black, Hispanic/Latino, or other. Clinical characteristics included primary cause of kidney failure (diabetes, hypertension, or other), treatment setting and modality (in-center hemodialysis, home dialysis, or preemptive transplantation), and, for hemodialysis patients, vascular access type (catheter or arteriovenous fistula).

To stratify by neighborhood socioeconomic disadvantage, we identified individuals living in neighborhoods in the top quintile of ADI, which reflects economic conditions for populations living in the most disadvantaged neighborhoods. We calculated county-level COVID-19 mortality rates by dividing the number of COVID-19 deaths from March 1, 2020, through June 30, 2020, by the county’s 2020 annual estimated population. More information about data sources is presented in eTable 1 in the Supplement.

### Statistical Analysis

We used Pearson χ^2^ tests to compare the sociodemographic and clinical characteristics of patients with incident kidney failure during the first 4 months of the COVID-19 pandemic (March to June 2020) to incident patients in the same 4-month time period in 2018 and 2019. We limited our comparisons to the same months before and during COVID-19 because of the seasonality of kidney failure incidence.^29^ We used *t* tests and Pearson χ^2^ tests to compare changes in eGFR, measured as continuous and categorical (less than 5, 5 to 10, 10 to 15, and greater than 15 mL/min/1.73 m^2^) variables, respectively. *P* values were 2-tailed and α < .05 was considered statistically significant.

To estimate the expected number of patients with incident kidney failure had the COVID-19 pandemic not occurred, we fitted a Poisson regression model using weekly counts of patients with incident kidney failure in the prepandemic period (January 2018 through February 2020), adjusting for linear time trend and seasonality using weekly fixed effects. We estimated counts of patients with incident kidney failure overall and separately for each racial or ethnic group. We then calculated change in kidney failure incidence during the pandemic, defined as the difference between the estimated and observed numbers of patients with incident kidney failure, and relative differences between estimated and observed patient counts. To assess disparities in the number of patients with incident kidney failure and kidney function at initiation, we stratified our analyses by patient race and ethnicity, residence in a county in the highest quintile of COVID-19 mortality rates, and residence in a socioeconomically disadvantaged neighborhood.^30,31,32^

## Results

### Characteristics of Patients With Incident Kidney Failure

In the pre–COVID-19 period (March through June of 2018 and 2019), 88 223 patients initiated treatment for incident kidney failure (mean [SD] age, 62.9 [15.3] years; 36 814 [41.7%] female, 22 157 [25.1%] non-Hispanic Black, and 13 398 [15.2%] Hispanic/Latino patients) (Table 1). During the first 4 months of the pandemic (March to June 2020), 38 924 patients initiated treatment for incident kidney failure (mean [SD] age, 62.6 [15.2] years, 16 225 [41.7%] female, 10 754 [27.6%] non-Hispanic Black, and 6440 [16.5%] Hispanic/Latino patients). Compared with the pre–COVID-19 period, in the first 4 months of the pandemic there were significant decreases in the proportion of patients with incident kidney failure who were non-Hispanic White (46 794 [53.0%] pre–COVID-19 vs 19 000 [48.8%] during COVID-19; *P* < .001), received a preemptive transplantation (1805 [2.1%] pre–COVID-19 vs 551 [1.4%] during COVID-19; *P* < .001), and initiated hemodialysis treatment with an arteriovenous fistula (12 098 [15.8%] pre–COVID-19 vs 4429 [13.4%] during COVID-19; *P* < .001). There were significant increases in the proportion of patients who were non-Hispanic Black (22 157 [25.1%] pre–COVID-19 vs 10 754 [27.6%] during COVID-19; *P* < .001), Hispanic/Latino (13 398 [15.2%] pre–COVID-19 vs 6440 [16.5%] during COVID-19; *P* < .001), initiating with home dialysis (10 690 [12.1%] pre–COVID-19 vs 5615 [14.4%] during COVID-19; *P* < .001), and initiating hemodialysis treatment with a catheter (61 837 [81.0%] pre–COVID-19 vs 27 805 [83.9%] during COVID-19; *P* < .001).

**Table 1.** Characteristics of Patients With Incident Kidney Failure, 2018-2020

Characteristic	Patients, No. (%)		*P* value
	2018-2019 (n = 88 218)^a^	2020 (n = 38 924)^a^	
Age, mean (SD), y	62.9 (15.3)	62.6 (15.2)	.01
Age category, y			
0-17	582 (0.7)	251 (0.6)	.05
18-44	10 228 (11.6)	4599 (11.8)	
45-64	32 588 (36.9)	14 567 (37.4)	
65-74	23 988 (27.2)	10 608 (27.3)	
75 and older	20 832 (23.6)	8899 (22.9)	
Sex			
Men	51 404 (58.3)	22 699 (58.3)	.88
Women	36 814 (41.7)	16 225 (41.7)	.88
Race or ethnicity			
Black	22 157 (25.1)	10 754 (27.6)	<.001
Hispanic/Latino	13 398 (15.2)	6440 (16.5)	
Other^b^	5869 (6.7)	2730 (7.0)	
White	46 794 (53.0)	19 000 (48.8)	
Current employment status			
Retired (age or disability)	51 636 (58.6)	23 129 (59.4)	<.001
Unemployed	20 709 (23.5)	8847 (22.7)	
Employed (full- or part-time)	11 517 (13.1)	4917 (12.6)	
Insurance type			
Dual Medicare and Medicaid	10 935 (12.4)	4657 (12.0)	<.001
Medicare	42 857 (48.6)	18 621 (47.8)	
Medicaid	13 033 (14.8)	5881 (15.1)	
Employer	11 152 (12.6)	5166 (13.3)	
Other	6041 (6.8)	2742 (7.0)	
Uninsured	4200 (4.8)	1857 (4.8)	
Primary cause			
Diabetes	41 994 (47.6)	18 365 (47.2)	.16
Hypertension	23 678 (26.8)	10 252 (26.3)	.06
Other	22 546 (25.6)	10 307 (26.5)	<.001
Treatment modality and setting			
In-center hemodialysis	74 925 (84.9)	32 534 (83.6)	<.001
Home dialysis	10 690 (12.1)	5615 (14.4)	<.001
Preemptive kidney transplantation	1805 (2.1)	551 (1.4)	<.001
Skilled nursing facility or long-term care facility dialysis	798 (0.9)	224 (0.6)	<.001
Received predialysis nephrology care			
Yes	60 592 (68.7)	26 887 (69.1)	<.001
No	15 131 (17.2)	6206 (15.6)	
Unknown	12 495 (14.2)	5831 (15.0)	
Vascular access type (hemodialysis patients)			
Catheter	61 837 (81.0)	27 805 (83.9)	<.001
Arteriovenous fistula	12 098 (15.8)	4429 (13.4)	
Other	2430 (3.2)	914 (2.7)	
eGFR, mean (SD), mL/min/1.73 m^2^	9.6 (5.0)	9.5 (4.9)	<.001
eGFR, mL/min/1.73 m^2^			
<5	11 999 (13.6)	5784 (14.9)	<.001
5-10	42 002 (47.7)	18 577 (47.8)	
10-15	24 553 (27.9)	10 553 (27.2)	
>15	9534 (10.8)	3954 (10.2)	

Abbreviation: eGFR, estimated glomerular filtration rate.

^a^ Samples limited to patients with incident kidney failure who initiated dialysis or received a preemptive transplantation between March 1 and June 30 of each year.

^b^ Other includes patients who are American Indian or Alaska Native, Asian, Native Hawaiian or Pacific Islander, and other race.

Among non-Hispanic White patients, there were significant increases in the proportion who had Medicare insurance (28 022 [59.9%] pre–COVID-19 vs 11 623 [61.2%] during COVID-19) and among Hispanic/Latino patients, there were significant increases in the proportion who were uninsured (1271 [9.5%] pre–COVID-19 vs 685 [10.6%] during COVID-19; *P* < .001) (Table 2). In the most disadvantaged neighborhoods (eTable 2 in the Supplement) and counties with the highest COVID-19 mortality rates (eTable 3 in the Supplement), there were significant decreases in the proportion of patients with incident kidney failure who were non-Hispanic White (eg, lowest ADI quintile neighborhoods: 6802 [47.9%] vs 2605 [44.6%]; *P* < .001) and significant increases in the proportion who were non-Hispanic Black.

**Table 2.** Characteristics of Patients With Incident Kidney Failure by Race and Ethnicity, 2018 to 2020

Characteristic	Non-Hispanic White			Non-Hispanic Black			Hispanic/Latino		
	Patients, No. (%)^a^		*P* value	Patients, No. (%)^a^		*P* value	Patients, No. (%)^a^		*P* value
	2018-2019 (n = 46 797)	2020 (n = 19 000)		2018-2019 (n = 22 158)	2020 (n = 10 755)		2018-2019 (n = 13 398)	2020 (n = 6440)	
Age, mean (SD), y	65.5 (14.6)	65.9 (14.2)	.003	60.0 (15.0)	59.6 (15.1)	.05	58.6 (15.9)	58.2 (15.6)	.10
Age category, y									
0-17	264 (0.6)	99 (0.5)	.02	123 (0.6)	71 (0.7)	.07	155 (1.2)	65 (1.0)	.04
18-44	3834 (8.2)	1434 (7.5)		3361 (15.2)	1696 (15.8)		2246 (16.8)	1123 (17.4)	
45-64	15 152 (32.4)	6069 (31.9)		9450 (42.7)	4554 (42.3)		5863 (43.8)	2917 (45.3)	
65-74	13 944 (29.8)	5809 (30.6)		5478 (24.7)	2722 (25.3)		3027 (22.6)	1354 (21.0)	
75 and older	13 600 (29.1)	5589 (29.4)		3745 (16.9)	1711 (15.9)		2107 (15.7)	981 (15.2)	
Sex									
Men	28 183 (60.2)	11 563 (60.9)	.14	11 949 (53.9)	5756 (53.5)	.49	7904 (59.0)	3825 (59.4)	.59
Women	18 611 (39.8)	7437 (39.1)		10 208 (46.1)	4998 (46.5)		5494 (41.0)	2615 (40.6)	
Current employment status									
Retired (age or disability)	31 192 (66.7)	13 146 (69.2)	<.001	11 620 (52.5)	5756 (53.6)	.02	5980 (44.7)	2842 (44.2)	.38
Unemployed	7878 (16.8)	2841 (15.0)		6471 (29.2)	3037 (28.3)		4750 (35.5)	2274 (35.3)	
Employed (full- or part-time)	5717 (12.2)	2209 (11.6)		3037 (13.7)	1404 (13.1)		1743 (13.0)	843 (13.1)	
Insurance type									
Dual	4485 (9.6)	1677 (8.8)	.002	3305 (14.9)	1630 (15.2)	.21	2212 (16.5)	943 (14.6)	<.001
Medicare	28 022 (59.9)	11 623 (61.2)		9081 (41.0)	4284 (39.8)		3758 (28.0)	1670 (27.5)	
Medicaid	4347 (9.3)	1679 (8.8)		3968 (17.9)	1974 (18.4)		3580 (26.7)	1670 (25.9)	
Employer	5683 (12.1)	2368 (12.5)		3069 (13.9)	1534 (14.3)		1520 (11.3)	795 (12.3)	
Other	2912 (6.3)	1163 (6.1)		1435 (6.5)	720 (6.7)		1057 (7.9)	578 (9.0)	
Uninsured	1345 (2.9)	490 (2.6)		1299 (5.9)	612 (5.7)		1271 (9.5)	685 (10.6)	
Primary cause									
Diabetes	21 326 (45.6)	8447 (44.5)	.01	9384 (42.4)	4534 (42.2)	.74	7974 (59.5)	3834 (59.5)	.98
Hypertension	11 299 (24.1)	4537 (23.9)	.47	8346 (37.7)	3877 (36.1)	.005	2726 (20.3)	1276 (19.8)	.38
Other	14 169 (30.3)	6016 (31.7)	<.001	4427 (20.0)	2343 (21.8)	<.001	2698 (20.1)	1330 (20.7)	.40
Treatment modality and setting									
In-center hemodialysis	39 064 (83.5)	15 539 (81.8)	<.001	19 410 (87.6)	9360 (87.0)	.15	11 575 (86.4)	5488 (85.2)	.03
Home dialysis	6011 (12.8)	2967 (15.6)	<.001	2240 (10.1)	1232 (11.5)	<.001	1555 (11.6)	863 (13.4)	<.001
Preemptive kidney transplantation	1316 (2.8)	393 (2.1)	<.001	197 (0.9)	69 (0.6)	.02	201 (1.5)	63 (1.0)	.003
Skilled nursing facility/long-term care facility dialysis	403 (0.9)	101 (0.5)	<.001	310 (1.4)	93 (0.9)	<.001	67 (0.5)	26 (0.4)	.35
Received predialysis nephrology care									
Yes	34 276 (73.2)	14 126 (74.3)	<.001	14 119 (63.7)	6829 (63.5)	<.001	8105 (60.5)	3963 (61.5)	.009
No	6721 (14.4)	2515 (13.2)		4301 (19.4)	1913 (17.8)		3126 (23.3)	1381 (21.4)	
Unknown	5797 (12.4)	2359 (12.4)		3737 (16.9)	2012 (18.7)		2167 (16.2)	1096 (17.0)	
Vascular access type (hemodialysis patients)									
Catheter	31 955 (80.1)	13 148 (82.8)	<.001	16 261 (81.7)	8091 (84.5)	<.001	9866 (84.4)	4828 (87.1)	<.001
Arteriovenous Fistula	6745 (16.9)	2335 (14.7)		2764 (13.9)	1103 (11.5)		1602 (13.7)	622 (11.2)	
Other	1174 (3.0)	387 (2.5)		879 (4.4)	379 (4.0)		224 (1.9)	96 (1.7)	
eGFR, mean (SD), mL/min/1.73 m^2^	10.4 (5.0)	10.4 (5.0)	.67	8.4 (4.6)	8.1 (4.5)	<.001	9.3 (4.9)	9.2 (4.9)	.25
eGFR, mL/min/1.73 m^2^									
<5	4227 (9.1)	1656 (8.7)	.39	4518 (20.4)	2534 (23.6)	<.001	2174 (16.3)	1117 (17.4)	.01
5-10	20 965 (44.9)	8525 (45.0)		11 705 (52.9)	5527 (51.4)		6358 (47.5)	3101 (48.2)	
10-15	15 255 (32.7)	6283 (33.1)		4388 (19.8)	2034 (18.9)		3559 (26.6)	1578 (24.5)	
>15	6262 (13.4)	2498 (13.2)		1522 (6.9)	652 (6.1)		1288 (9.6)	634 (9.9)	

Abbreviation: eGFR, estimated glomerular filtration rate.

^a^ Samples limited to patients with incident kidney failure who initiated dialysis or received a preemptive transplantation between March 1 and June 30 of each year.

### Changes in Number of Patients With Incident Kidney Failure

**Figure 1.**
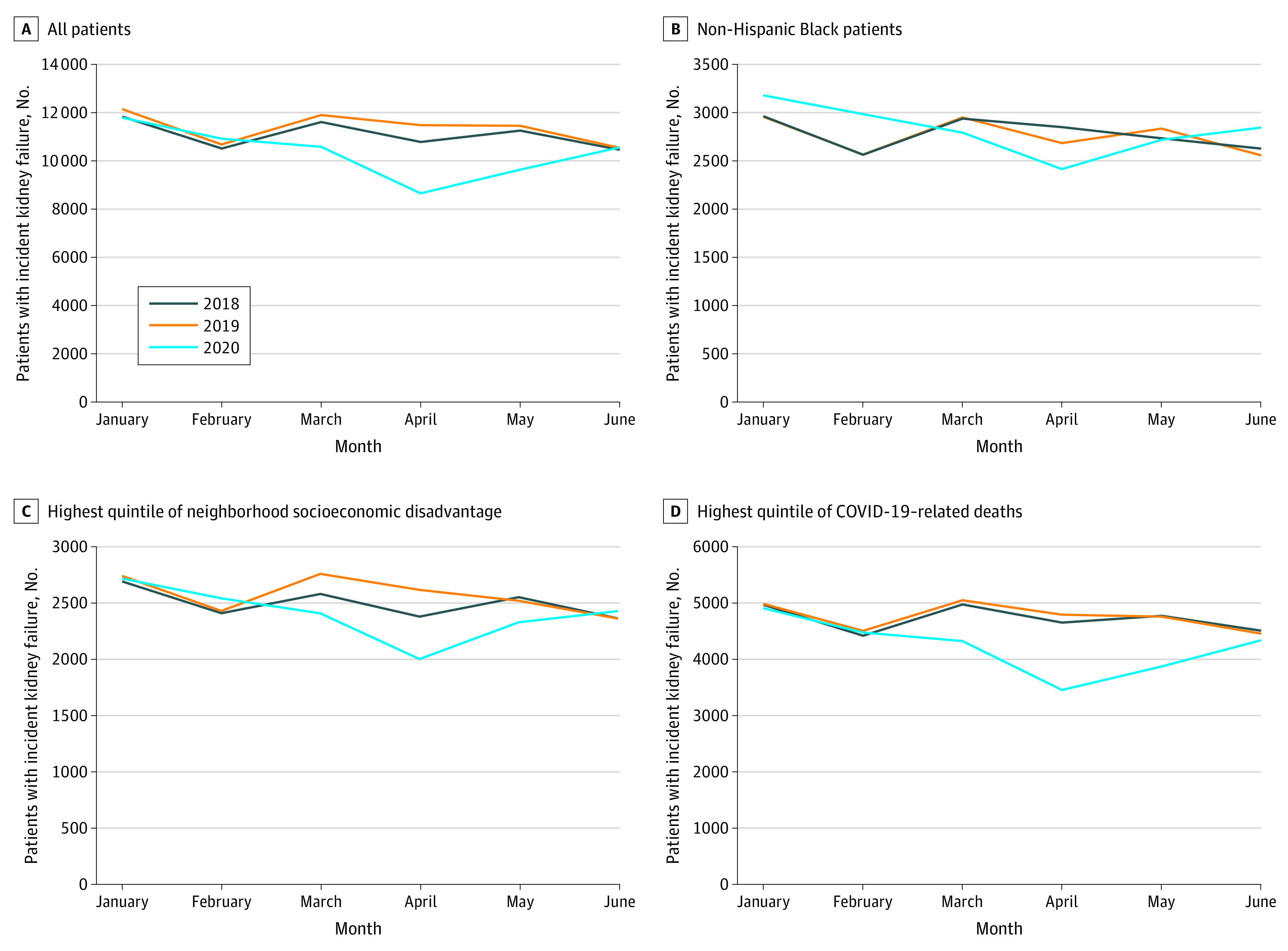
Monthly Trends in Number of Patients With Incident Kidney Failure, January 2018 to June 2020 Census block groups were separated into quintiles of area deprivation based on scores in the 2018 Area Deprivation Index. Highest quintile of neighborhood disadvantage reflects the most disadvantaged neighborhoods. Quintiles of county-level COVID-19–related deaths per capita between March 1 and June 30, 2020, were calculated using the daily number of new COVID-19–related deaths reported by the Centers of Disease Control and county population estimates from the US Census Bureau. Because of the number of deaths that were indicated as “Unallocated/Probable” in New York City, we combined the number of COVID-19–related deaths and populations in the 5 counties that comprise New York City (ie, Bronx, Kings, New York, Queens, and Richmond).

Figure 1 displays nationwide monthly trends in the number of patients with incident kidney failure from January 2018 through June 2020. The COVID-19 pandemic coincided with a nationwide decrease in the number of patients initiating treatment for kidney failure, with the largest reduction observed in April 2020 compared with the prepandemic time period. The number of patients initiating treatment began to gradually rebound in May and June 2020. There were reductions in the number of patients with incident kidney failure initiating treatment between March and April 2020 for non-Hispanic White, non-Hispanic Black, and Hispanic/Latino patients (eFigure 1 in the Supplement).

### Changes in Kidney Function at Treatment Initiation

**Figure 2.**
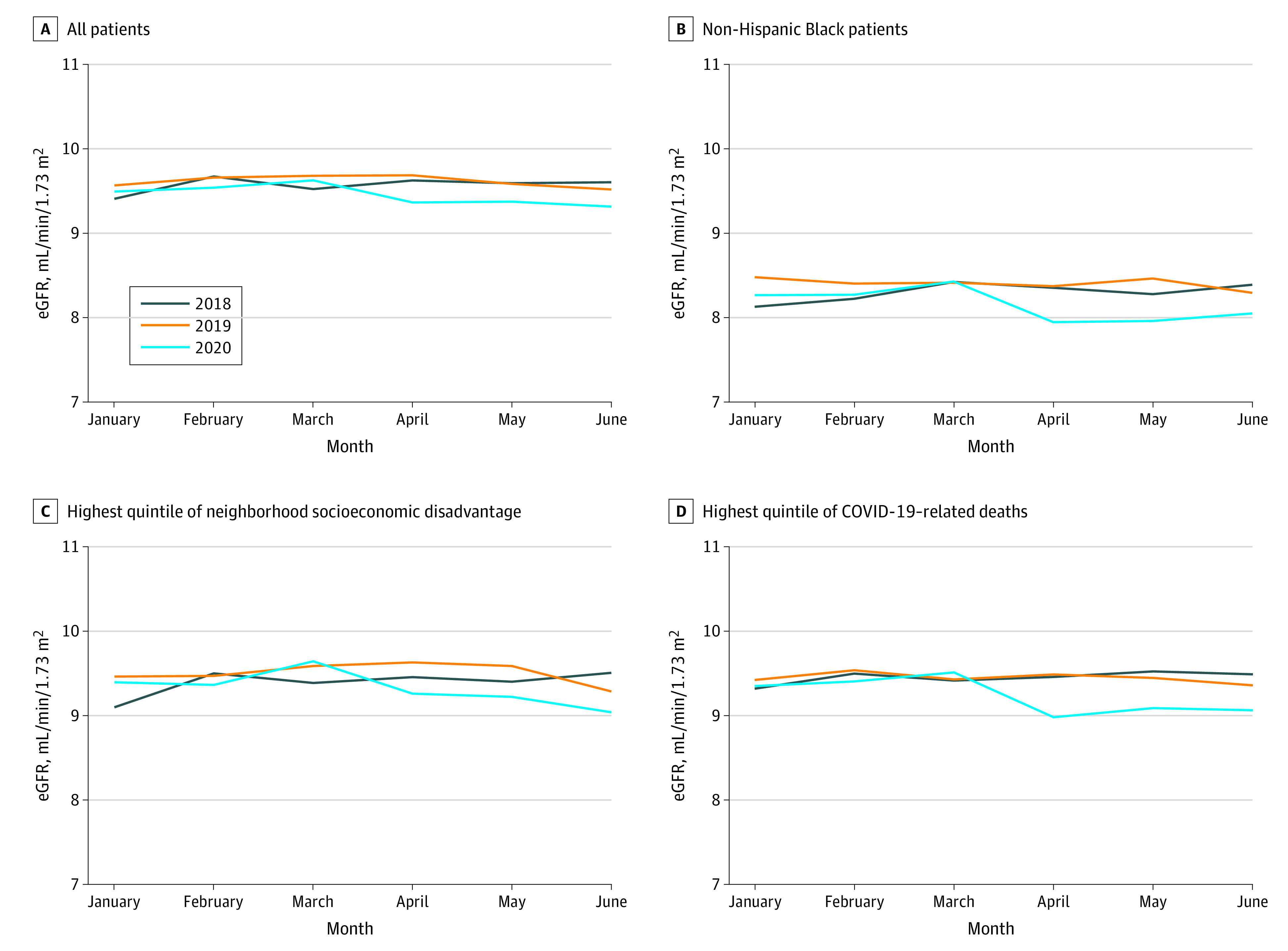
Trends in Mean Estimated Glomerular Filtration Rate (eGFR) at Treatment Initiation, January 2018 to June 2020 Census block groups were separated into quintiles of area deprivation based on scores in the 2018 Area Deprivation Index. Highest quintile of neighborhood disadvantage reflects the most disadvantaged neighborhoods.Quintiles of county-level COVID-19–related deaths per capita between March 1 and June 30, 2020, were calculated using the daily number of new COVID-19–related deaths reported by the Centers of Disease Control and county population estimates from the US Census Bureau. Because of the number of deaths that were indicated as “Unallocated/Probable” in New York City, we combined the number of COVID-19–related deaths and populations in the 5 counties that comprise New York City (Bronx, Kings, New York, Queens, and Richmond).

Figure 2 presents nationwide monthly trends in mean eGFR at treatment initiation. The pre–COVID-19 mean (SD) eGFR at treatment initiation was 9.6 (5.0) mL/min/1.73 m^2^, which declined to 9.5 (4.9) mL/min/1.73 m^2^ during the pandemic (*P* < .001) (Table 1; Figure 2). Unlike the gradual rebound observed in the number of patients with incident kidney failure, the mean eGFR remained lower than before the pandemic throughout the study period. Reductions in mean eGFR were exclusively observed among non-Hispanic Black patients (8.4 [4.6] mL/min/1.73 m^2^ pre–COVID-19 vs 8.1 [4.5] mL/min/1.73 m^2^ during COVID-19; *P* < .001), with significantly more initiating treatment with eGFR lower than 5 mL/min/1.73 m^2^ (4520 [20.4%] pre–COVID-19 vs 2538 [23.6%] during COVID-19; *P* < .001) (Table 2; Figure 2). There were not statistically significant changes in mean eGFR for non-Hispanic White or Hispanic/Latino patients (Table 2; eFigure 2in the Supplement). There were significant decreases in eGFR among patients across all quintiles of neighborhood disadvantage and among patients residing in counties with the highest COVID-19 mortality rates (9.5 [5.0] mL/min/1.73 m^2^ pre–COVID-19 vs 9.2 [4.9] mL/min/1.73 m^2^ during COVID-19; *P* < .001), but no significant difference for patients residing in lower-COVID-19 counties. (Figure 2; eTables 2 and 3 and eFigure 2 in the Supplement).

### Estimated Changes in Kidney Failure Incidence

**Figure 3.**
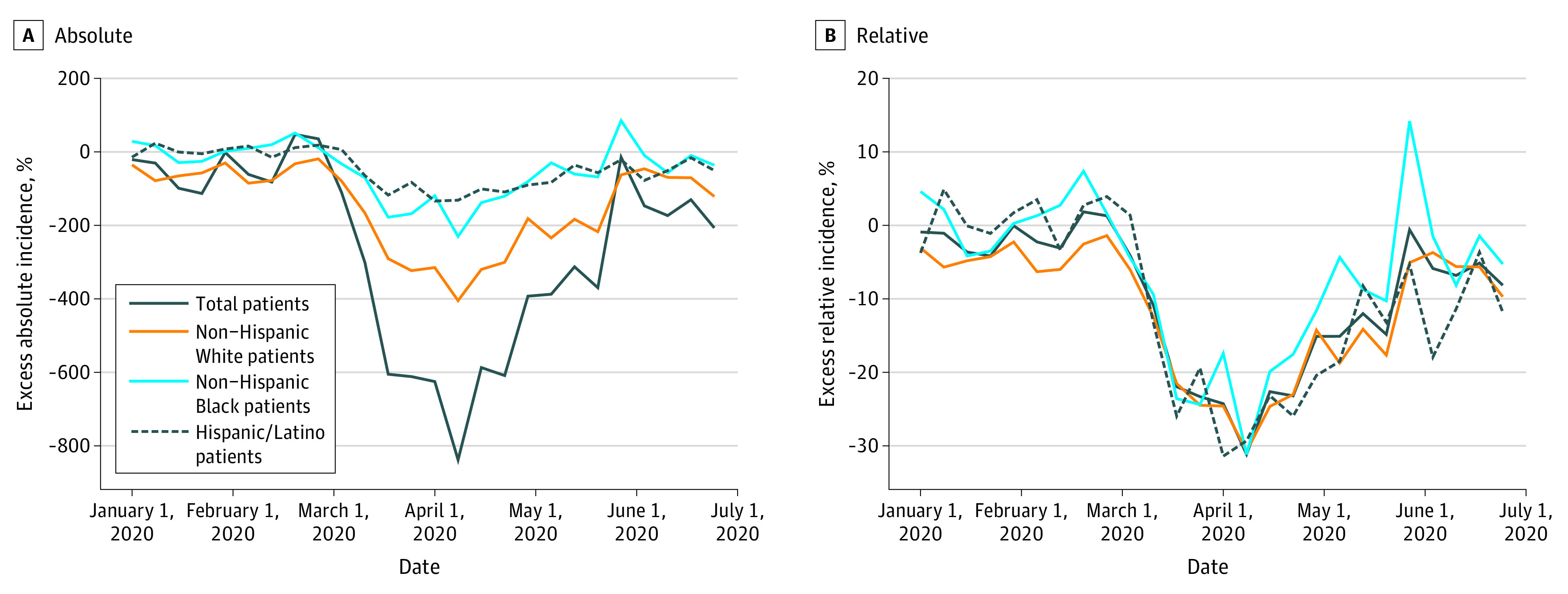
Changes in Kidney Failure Incidence by Week and Race and Ethnicity, January to June 2020 The estimated number of patients with incident kidney failure was estimated by fitting a Poisson regression model using weekly counts of the number of patients with incident kidney failure in the prepandemic period (January 2018 through February 2020), adjusting for linear time trend and seasonality using weekly fixed effects. Counts were estimated overall and separately for each racial/ethnic group. Change in kidney failure incidence was defined as the difference between the estimated and observed numbers of patients with incident kidney failure, and relative differences as between the estimated and observed counts.

The substantial overlap between observed and estimated number of patients with incident kidney failure by week indicates that the Poisson model fit the data well and provides reliable estimates for the first 4 months of the pandemic (eFigures 3 and 4 in the Supplement). Figure 3 presents the changes in kidney failure incidence by week in absolute and relative terms, both overall and by race and ethnicity. Compared with pre–COVID-19 trends, the total number of patients with incident kidney failure nationwide declined substantially in the second week of April 2020 (absolute change, −800 patients; relative change, −30%). The total number of patients with incident kidney failure gradually rebounded in the third and fourth months of the pandemic but did not reach estimated levels based on historical trends. All racial and ethnic groups experienced large relative reductions of approximately 30% in the number of patients with incident kidney failure in April 2020.

## Discussion

Compared with trends in prior years, there was a steep decline in the number of people initiating treatment for kidney failure during the first 4 months of the COVID-19 pandemic. Among patients who initiated treatment for kidney failure during the early months of the pandemic, there was a decline in mean eGFR at treatment initiation—particularly evident among non-Hispanic Black patients and patients in counties with high COVID-19 mortality—indicating that treatment was initiated at lower levels of kidney function. Patients who initiated treatment for kidney failure during the pandemic were less likely to receive a preemptive transplantation and to initiate hemodialysis using an arteriovenous fistula for vascular access than incident patients in the prepandemic period.

The significant decline in the number of patients with incident kidney failure in the early months of the COVID-19 pandemic, which is consistent with US Renal Data System’s estimates,^33^ may be due to several factors. Some patients may have delayed treatment initiation, but others may have died before being able to initiate treatment. Although the number of incident patients rebounded during the study period, the number of patients with incident kidney failure still did not reach the estimated level based on historical trends even during the third and fourth months of the pandemic. Several mechanisms may have concurrently contributed to reduced treatment initiation—including limited appointment availability, fear of infection, shared patient-provider decisions to defer treatment initiation, and financial barriers to accessing care^4^—and to lower mean kidney function at initiation, such as delayed care, food insecurity, and inability to manage kidney care.^30^ While this study was not designed to empirically assess these mechanisms in this data, this warrants further exploration. Importantly, the reduction in treatment initiation could reflect higher mortality among patients with CKD, whether because of COVID-19 infection or another cause.^32,34^

It is possible that our findings reflect changes in kidney care delivery in response to the pandemic, rather than delayed treatment initiation for CKD patients. In efforts to mitigate COVID-19 transmission and conserve personal protective equipment, there was limited access to elective procedures. Our findings support other reports of delayed AVF placement during the pandemic.^35^ Some studies suggest that initial CMS guidance to defer nonessential surgical procedures may have led to delays, despite clarification that dialysis access procedures are essential.^35^ Considering AVF planning and maturation can take between 6 and 12 months, the observed declines may not exclusively be COVID-19–related, and continued monitoring of trends in timely vascular access procedures later into the pandemic is critical. Other surgical procedures, such as preemptive transplantations, were also limited early in the pandemic.^36^ It is possible that the decline in preemptive transplantation may reflect concerns of increased susceptibility to COVID-19 among immunosuppressed transplantation recipients.^37^ Furthermore, for some patients, late-stage CKD care was likely converted to telehealth early in the pandemic.^6,35^ Our findings suggest that routine care may have nevertheless been missed, perhaps because of barriers to or suboptimal quality of telehealth for late-stage CKD patients, particularly for Black patients. More broadly, while kidney replacement therapy is necessary for many patients with kidney failure, its initiation is subjective. Declines in number of patients initiating treatment for kidney failure likely suggest a combination of delays in necessary care, clinically reasonable delays in treatment agreed upon by patients with CKD and their doctors, delays in treatment initiation for patients who may have otherwise started therapy earlier than would be optimal, and potentially higher mortality rates among patients with advanced CKD.

Our findings suggest that during the pandemic patients with incident kidney failure initiated treatment with statistically lower eGFR levels than pre–COVID-19. The relationship between eGFR at treatment initiation, optimal timing of treatment initiation, and outcomes remains unclear, and some patients may initiate treatment earlier than is optimal. It is possible that the observed decline in eGFR is not clinically meaningful, although it will be critical to evaluate the long-term outcomes of individuals initiating treatment with lower eGFR levels during the pandemic. Furthermore, this decline was exclusively seen in non-Hispanic Black patients. The Chronic Kidney Disease Epidemiology Collaboration equation widely used to measure kidney function includes a modifier for Black race, thereby systematically raising eGFR for Black individuals by 16%.^26^ There are ongoing debates regarding inclusion of race in the equation because race is a social rather than biological construct, and so racial adjustments to eGFR could potentially perpetuate systemic inequities in care delivery (eg, delayed diagnosis, suboptimal preventive care, and decreased eligibility for nephrology specialty care) among Black patients with CKD.^25,38^

Our study has several implications for policy and practice. First, it remains critical to maintain continuity of care and improve care delivery for people with CKD. For example, the Advancing American Kidney Health Initiative and CMS have prioritized increasing home dialysis and transplantation as a central strategy in improving kidney care. Improving access to care and reducing disparities for people with kidney disease are national priorities, but our findings suggest that the early months of the COVID-19 pandemic were associated with lower rates of treatment initiation for this high-risk population with social risk factors. Second, state and federal policies may play a critical role in dually reducing cost-related barriers to care and unmet social needs. For example, in states that have not done so, expanding Medicaid eligibility could facilitate access to care for low-income patients with incident kidney failure and reduce kidney failure incidence.^39,40,41^ Other policy reforms to expand eligibility and access to social safety net programs (eg, extending federal unemployment compensation) may mitigate unmet social needs and prevent disease progression.^42,43^

### Limitations

Our study has several limitations. First, it is possible that data reported by dialysis facilities and transplantation clinics during the first half of 2020 is incomplete. Data after June 2020 were not available, and continued evaluation of changes in treatment initiation later into the pandemic is important. Second, our analysis is unable to examine specific mechanisms to explain reduced treatment initiation or account for the discontinuation of dialysis or competing risk of death, whether due to untreated kidney failure, COVID-19, or another cause. Third, because area-level population estimates are not yet available for 2020, we were unable to calculate changes in the rate of kidney failure incidence and instead modeled counts of incident patients.

## Conclusions

This cross-sectional study of US adults identified substantial declines in the number of patients with incident kidney failure initiating treatment in the first 4 months of the COVID-19 pandemic, and found patients initiated treatment with significantly lower kidney function. Initiation of kidney replacement therapy with worsened kidney function was particularly evident among Black patients and those living in counties with higher COVID-19 mortality rates. This suggests that the COVID-19 pandemic was associated with delayed treatment for kidney failure, a life-threatening condition that disproportionately impacts socially disadvantaged populations in the US. The implications of these findings for disparities in CKD warrant further investigation.

## Notes

Supplement. **eTable 1.** Measure Definitions Data Sources **eTable 2.** Characteristics of Patients with Incident Kidney Failure by Quintile of Neighborhood Area Disadvantage, 2018-2020 **eTable 3.** Characteristics of Patients with Incident Kidney Failure by Residence in a County COVID-19 Mortality Rate Quintile, 2018-2020 **eFigure 1.** Monthly Trends in Number of Incident Kidney Failure Patients by Race/Ethnicity, Neighborhood Socioeconomic Disadvantage, and County-Level COVID-19 Mortality Rate, January 2018-June 2020 **eFigure 2.** Trends in Mean Estimated Glomerular Filtration Rate (eGFR) at Treatment Initiation by Race/Ethnicity, Neighborhood Disadvantage, and COVID-19 Mortality Rate, January 2018-June 2020 **eFigure 3.** Comparison of Observed and Estimated Number of Patients with Incident Kidney Failure by Week, January 2018-June 2020 **eFigure 4.** Changes in Kidney Failure Incidence by Week and Race/Ethnicity, January 2018-June 2020
eReferences
