# Supplementary material for: Evaluation of Racial, Ethnic, and Socioeconomic Disparities in Initiation of Kidney Failure Treatment During the First 4 Months of the COVID-19 Pandemic
Source: JAMA Netw Open. 2021 Oct 7;4(10):e2127369. doi: 10.1001/jamanetworkopen.2021.27369 (PMC8498850; doi:10.1001/jamanetworkopen.2021.27369)
Supplement: Supplement. — eTable 1. Measure Definitions Data Sources eTable 2. Characteristics of Patients with Incident Kidney Failure by Quintile of Neighborhood Area Disadvantage, 2018-2020 eTable 3. Characteristics of Patients with Incident Kidney Failure by Residence in a County COVID-19 Mortality Rate Quintile, 2018-2020 eFigure 1. Monthly Trends in Number of Incident Kidney Failure Patients by Race/Ethnicity, Neighborhood Socioeconomic Disadvantage, and County-Level COVID-19 Mortality Rate, January 2018-June 2020 eFigure 2. Trends in Mean Estimated Glomerular Filtration Rate (eGFR) at Treatment Initiation by Race/Ethnicity, Neighborhood Disadvantage, and COVID-19 Mortality Rate, January 2018-June 2020 eFigure 3. Comparison of Observed and Estimated Number of Patients with Incident Kidney Failure by Week, January 2018-June 2020 eFigure 4. Changes in Kidney Failure Incidence by Week and Race/Ethnicity, January 2018-June 2020 eReferences [file jamanetwopen-e2127369-s001.pdf]

Supplemental Online Content

Nguyen KH, Thorsness R, Hayes S, et al. Evaluation of racial, ethnic, and socioeconomic disparities in initiation of kidney failure treatment during the first 4 months of the COVID-19 pandemic. *JAMA Netw Open.* 2021;4(10):e2127369.
doi:10.1001/jamanetworkopen.2021.27369

eTable 1. Measure Definitions Data Sources

eTable 2. Characteristics of Patients with Incident Kidney Failure by Quintile of Neighborhood Area Disadvantage, 2018-2020

eTable 3. Characteristics of Patients with Incident Kidney Failure by Residence in a County COVID-19 Mortality Rate Quintile, 2018-2020

eFigure 1. Monthly Trends in Number of Incident Kidney Failure Patients by Race/Ethnicity, Neighborhood Socioeconomic Disadvantage, and County-Level COVID-19 Mortality Rate, January 2018-June 2020

eFigure 2. Trends in Mean Estimated Glomerular Filtration Rate (eGFR) at Treatment Initiation by Race/Ethnicity, Neighborhood Disadvantage, and COVID-19 Mortality Rate, January 2018-June 2020

eFigure 3. Comparison of Observed and Estimated Number of Patients with Incident Kidney Failure by Week, January 2018-June 2020

eFigure 4. Changes in Kidney Failure Incidence by Week and Race/Ethnicity, January 2018-June 2020

eReferences

This supplemental material has been provided by the authors to give readers additional information about their work.

© 2021 Nguyen KH et al. *JAMA Network Open.*

eTable 1. Measure Definitions Data Sources

Variable	Data Source(s)
Incident kidney failure cases	End-Stage Renal Disease Medical Evidence Report (CMS 2728)[1]
Stratified Analyses	
Race/Ethnicity	End-Stage Renal Disease Medical Evidence Report (CMS 2728)[1]
Residing in a socioeconomically disadvantaged neighborhood (highest quintile)	Area Deprivation Index, V3.0[2,3] using the Census Block Group-level measure
Residing in a county with high COVID-19 mortality rate (highest quintile)	Numerator: Number of COVID-19-related deaths, March 1, 2020-June 30,2020[4]<br><br>Denominator: U.S. Census Bureau County Annual Population Estimates, 2020[5]

© 2021 Nguyen KH et al. *JAMA Network Open.*

eTable 2. Characteristics of Patients with Incident Kidney Failure by Quintile of Neighborhood Area Disadvantage, 2018-2020 [a,b]

Characteristic	Lowest Quintile of Neighborhood Socioeconomic Disadvantage			Highest Quintile of Neighborhood Socioeconomic Disadvantage		
	2018-2019	2020	p	2018-2019	2020	p
N	14,201	5,840		20,135	9,169	
Age, mean (SD)	64.9 (15.7)	64.7 (15.3)	0.44	61.2 (14.8)	60.9 (14.9)	0.040
Age Category, %						
0-17	0.7	0.7	0.13	0.5	0.6	0.21
18-44	10.0	9.7		13.2	13.7	
45-64	33.3	33.8		41.1	41.2	
65-74	26.1	27.5		26.3	26.3	
75 and older	29.9	28.3		18.9	18.1	
Male	62.2	61.7	0.59	53.9	54.5	0.37
Female, %	37.8	38.3	0.59	46.1	45.5	0.37
Race/Ethnicity, %						
White	47.9	44.6	<0.001	44.0	37.9	<0.001
Black	15.4	16.1	0.19	39.3	43.5	<0.001
Hispanic/Latino	18.7	19.0	0.66	13.3	15.1	<0.001
Current Employment Status, %						
Retired (Age or Disability)	56.6	57.9	0.39	57.4	58.0	0.007
Unemployed	21.3	20.9		28.9	27.5	
Employed (Full/Part)	16.4	15.8		9.4	9.5	
Insurance Type, %						
Dual	12.1	10.9	<0.001	16.4	16.2	0.054
Medicare	45.5	45.9		44.6	43.1	
Medicaid	16.6	16.8		16.7	17.4	
Employer	14.7	16.3		9.6	9.7	
Other	8.4	8.6		5.0	5.6	
Uninsured	2.7	1.5		6.9	7.4	
Primary Cause, %						
Diabetes	45.5	45.9	0.53	50.7	48.6	0.001
Hypertension	25.5	24.1	0.047	28.6	29.0	0.52
Other	29.1	29.9	0.23	20.7	22.4	<0.001
Treatment Modality & Setting, %						
In-Center Hemodialysis	82.3	80.3	<0.001	88.4	87.8	0.16
Home Dialysis	13.7	16.8	<0.001	10.0	11.1	0.006
Preemptive Kidney Transplant	3.4	2.4	<0.001	0.9	0.6	0.009
Skilled Nursing Facility/Long-Term Care Facility Dialysis	0.6	0.4	0.1	0.7	0.5	0.05
Received Pre-Dialysis Nephrology Care, %						
Yes	68.7	69.7	0.13	66.8	65.1	<0.001
No	15.9	14.8		18.7	18.6	
Unknown	15.4	15.5		14.5	16.3	
Vascular Access Type (Hemodialysis Patients), %						
Catheter	77.7	80.8	<0.001	82.9	85.9	<0.001
Arteriovenous Fistula	18.9	16.1		13.7	11.4	
Other	3.4	3.1		3.4	2.7	

© 2021 Nguyen KH et al. *JAMA Network Open.*

eGFR (mL/min/1.73m^2), mean (SD)	9.4 (5.0)	9.2 (5.0)	0.001	9.5 (4.9)	9.3 (4.9)	0.002
eGFR (mL/min/1.73m^2), %						
<5	14.9	16.6	0.003	14.7	16.4	0.001
5-10	48.8	49.0		47.9	47.9	
10-15	25.8	25.0		26.9	25.5	
>15	10.5	9.4		10.5	10.3	

Footnotes: [a] Samples limited to incident kidney failure patients who initiated dialysis or had a preemptive transplant between March 1 and June 30 of each year. [b]Census block groups were separated into quintiles of area deprivation based on scores in the 2018 Area Deprivation Index.

© 2021 Nguyen KH et al. *JAMA Network Open.*

eTable 3. Characteristics of Patients with Incident Kidney Failure by Residence in a County COVID-19 Mortality Rate Quintile, 2018-2020 [a,b]

Characteristic	Lowest County-Level COVID-19 Mortality Rate Quintile			Highest County-Level COVID-19 Mortality Rate Quintile		
	2018-2019	2020	p	2018-2019	2020	p
N	4,940	2,217		37,970	15,992	
Age, mean (SD)	63.5 (14.7)	64.0 (14.7)	0.21	63.3 (15.4)	62.8 (15.2)	<0.001
Age Category, %						
0-17	0.5	0.8	0.34	0.6	0.6	0.002
18-44	10.5	9.9		11.3	11.7	
45-64	34.9	33.8		36.3	37.5	
65-74	29.7	31.2		26.9	26.9	
75 and older	24.4	24.4		24.9	23.3	
Male	58.7	58.4	0.84	58.6	58.6	0.89
Female, %	41.3	41.6	0.84	41.4	41.4	0.89
Race/Ethnicity, %						
White	78.9	76.4	0.020	47.5	43.0	<0.001
Black	8.1	8.7	0.36	31.0	35.3	<0.001
Hispanic/Latino	7.6	8.7	0.11	15.0	14.9	0.87
Current Employment Status, %						
Retired (Age or Disability)	66.7	68.7	0.30	58.0	58.2	0.31
Unemployed	17.8	17.5		24.1	23.5	
Employed (Full/Part)	11.1	10.1		12.8	12.9	
Insurance Type, %						
Dual	12.2	13.2	0.25	14.0	13.3	<0.001
Medicare	56.3	54.7		46.3	45.4	
Medicaid	11.6	11.8		17.3	18.0	
Employer	9.8	8.8		12.6	13.7	
Other	5.5	5.9		5.8	5.9	
Uninsured	0.8	0.7		0.4	0.4	
Primary Cause, %						
Diabetes	51.1	48.8	0.07	45.6	45.2	0.47
Hypertension	20.1	20.8	0.50	28.9	28.1	0.064
Other	28.8	30.4	0.17	25.5	26.7	0.006
Treatment Modality & Setting, %						
In-Center Hemodialysis	84.4	81.9	0.009	85.6	84.6	0.004
Home Dialysis	13.3	17.1	<0.001	10.6	12.8	<0.001
Preemptive Kidney Transplant	2.0	0.9	<0.001	2.1	1.5	<0.001
Skilled Nursing Facility/Long-Term Care Facility Dialysis	0.2	0.2	<0.001	1.7	1.1	<0.001
Received Pre-Dialysis Nephrology Care, %						
Yes	73.9	73.8	0.44	66.3	66.8	<0.001
No	16.6	15.8		17.5%	15.8%	
Unknown	9.5	10.3		16.2%	17.3%	
Vascular Access Type (Hemodialysis Patients), %						
Catheter	82.5	84.3	0.29	81.3	84.1	<0.001
Arteriovenous Fistula	15.4	14.0		15.3	13.1	
Other	2.1	1.7		3.5	2.8	
eGFR (mL/min/1.73m^2), mean (SD)	10.3 (4.9)	10.3 (5.0)	0.81	9.5 (5.0)	9.2 (4.9)	<0.001

© 2021 Nguyen KH et al. *JAMA Network Open.*

eGFR (mL/min/1.73m^2), %						
<5	12.8	13.2	0.02	14.8	17.2	<0.001
5-10	47.3	47.8		48.2	47.8	
10-15	28.9	28.5		26.5	25.2	
>15	11.1	10.5		10.5	9.8	

Footnotes: [a] Samples limited to incident kidney failure patients who initiated dialysis or had a preemptive transplant between March 1 and June 30 of each year. [b] Quintiles of county-level COVID-19-related deaths per capita calculated using county-level daily number of new COVID-19-related deaths (March-June 2020) and U.S. Census Bureau county population estimates for 2020.

© 2021 Nguyen KH et al. *JAMA Network Open.*

eFigure 1. Monthly Trends in Number of Incident Kidney Failure Patients by Race/Ethnicity, Neighborhood Socioeconomic Disadvantage, and County-Level COVID-19 Mortality Rate, January 2018-June 2020

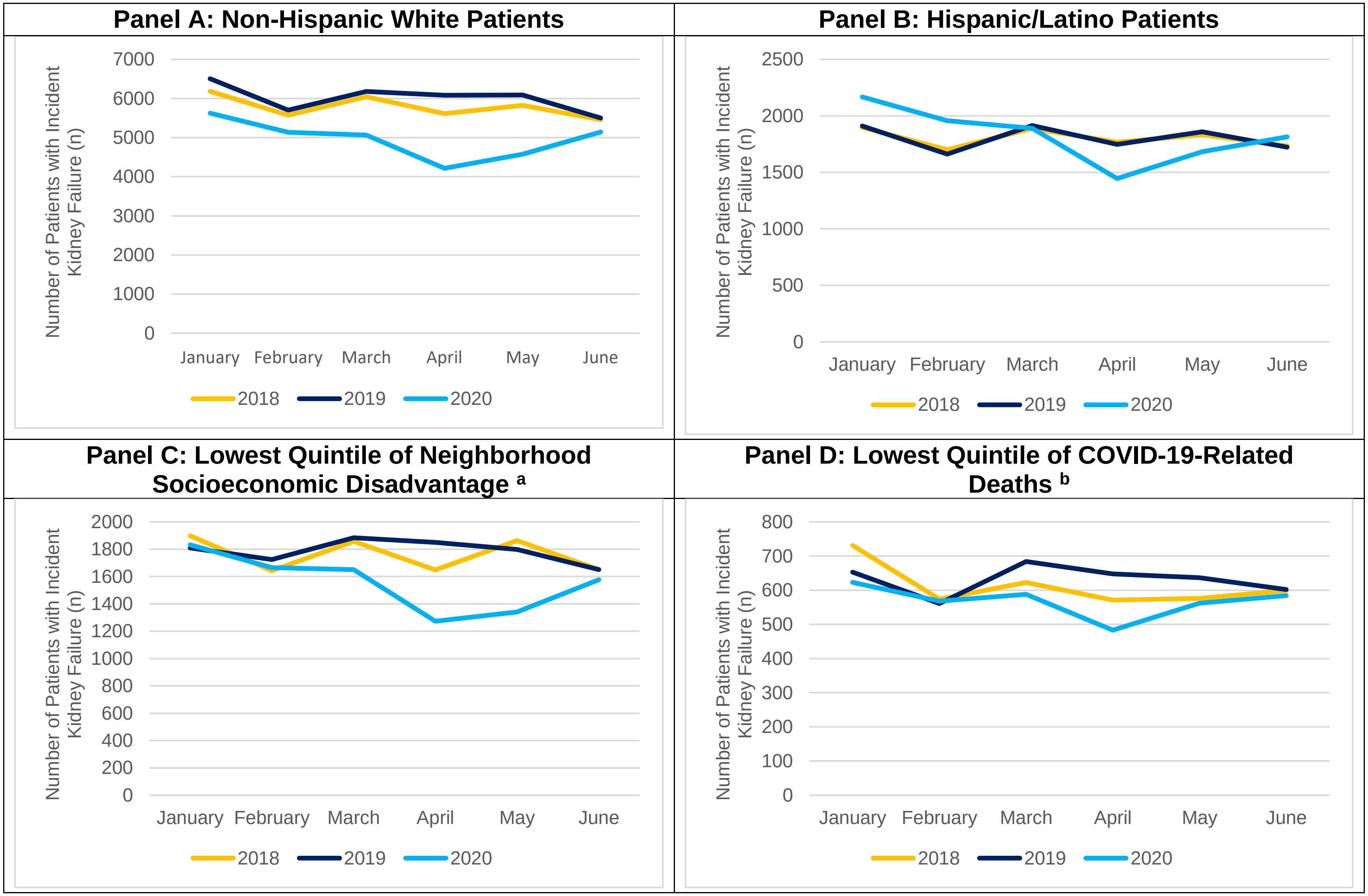

© 2021 Nguyen KH et al. *JAMA Network Open.*

Footnotes. [a] Census block groups were separated into quintiles of area deprivation based on scores in the 2018 Area Deprivation Index. "Lowest Quintile of Neighborhood Disadvantage" reflects the least disadvantaged neighborhoods. [b] Quintiles of county-level COVID-19-related deaths per capita between March 1-June 30, 2020 were calculated using CDC-reported daily number of new COVID-19-related deaths, and denominator used U.S. Census Bureau county population estimates. Because of the number of deaths that were indicated as "Unallocated/Probable" in New York City, we combined the number of COVID-19-related deaths and populations in the five counties that comprise New York City (Bronx, Kings, New York, Queens, and Richmond).

© 2021 Nguyen KH et al. *JAMA Network Open.*

eFigure 2. Trends in Mean Estimated Glomerular Filtration Rate (eGFR) at Treatment Initiation by Race/Ethnicity, Neighborhood Disadvantage, and COVID-19 Mortality Rate, January 2018-June 2020

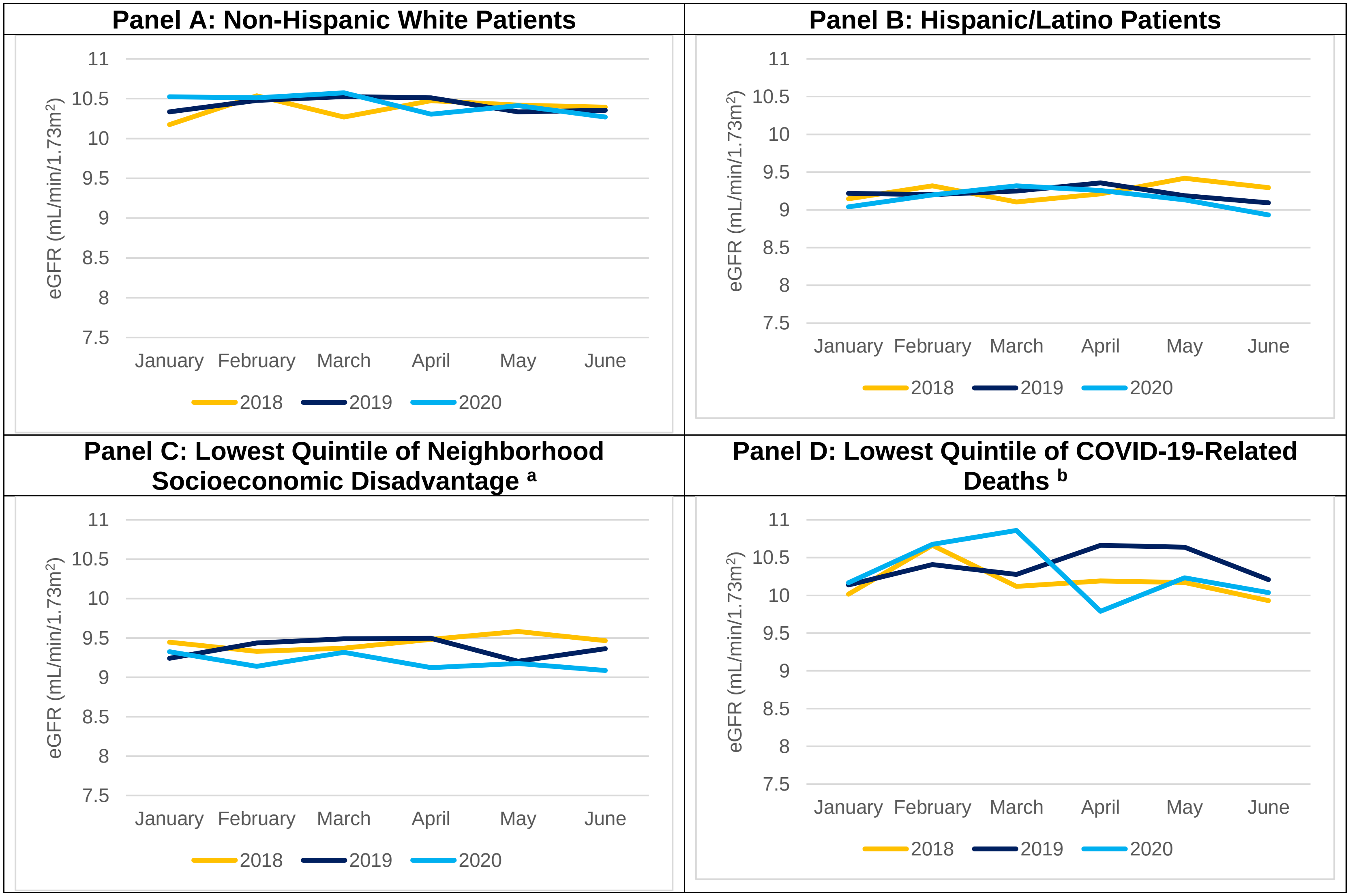

© 2021 Nguyen KH et al. *JAMA Network Open.*

Footnotes. [a] Census block groups were separated into quintiles of area deprivation based on scores in the 2018 Area Deprivation Index. "Lowest Quintile of Neighborhood Disadvantage" reflects the least disadvantaged neighborhoods. [b] Quintiles of county-level COVID-19-related deaths per capita between March 1-June 30, 2020 were calculated using CDC-reported daily number of new COVID-19-related deaths, and denominator used U.S. Census Bureau county population estimates. Because of the number of deaths that were indicated as "Unallocated/Probable" in New York City, we combined the number of COVID-19-related deaths and populations in the five counties that comprise New York City (Bronx, Kings, New York, Queens, and Richmond).

© 2021 Nguyen KH et al. *JAMA Network Open.*

eFigure 3. Comparison of Observed and Estimated Number of Patients with Incident Kidney Failure by Week, January 2018-June 2020 [a]

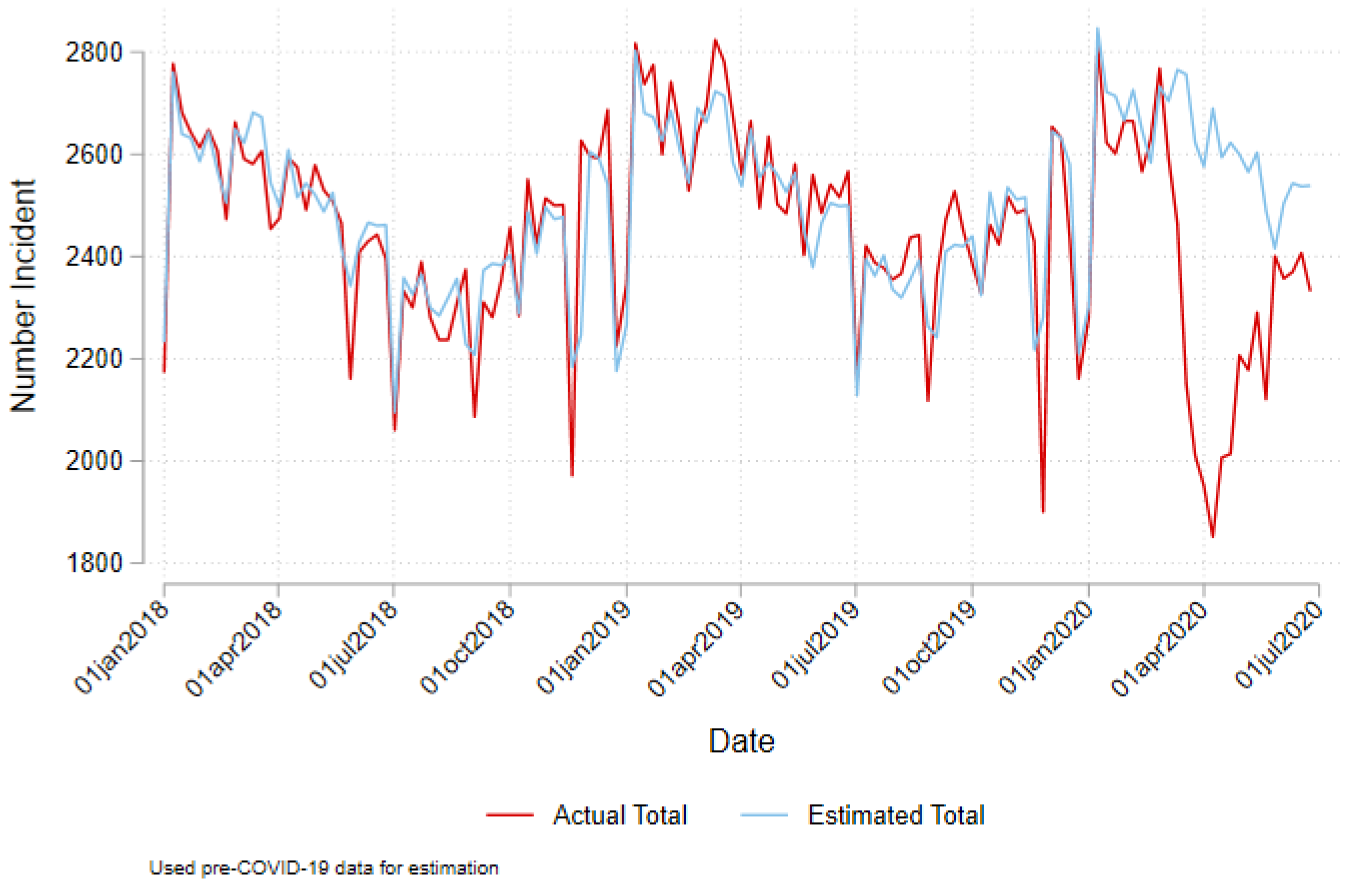

Footnotes. [a] The estimated number of patients with incident kidney failure patients was estimated by fitting a Poisson regression model using weekly counts of the number of patients with incident kidney failure in the pre-pandemic period (January 2018 through February 2020), adjusting for linear time trend and seasonality using weekly fixed effects.

© 2021 Nguyen KH et al. *JAMA Network Open.*

eFigure 4. Changes in Kidney Failure Incidence by Week and Race/Ethnicity, January 2018-June 2020 [a]

Panel A: Absolute	Panel B: Relative

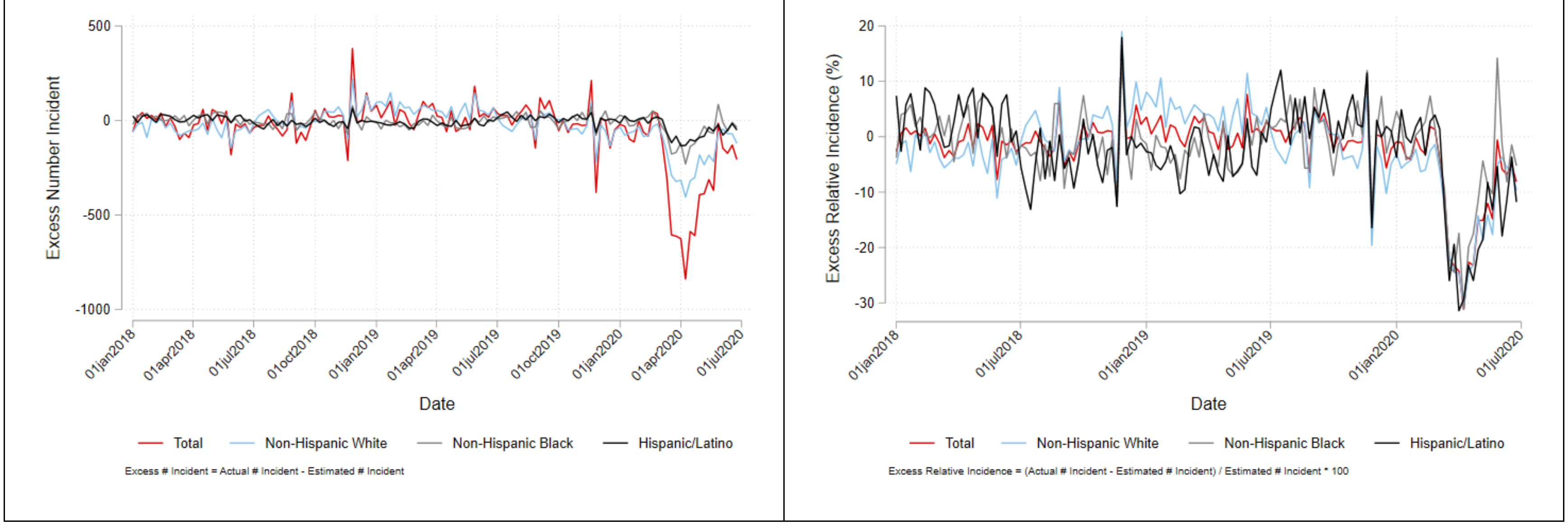

Footnotes. [a] The estimated number of patients with incident kidney failure was estimated by fitting a Poisson regression model using weekly counts of the number of patients with incident kidney failure in the pre-pandemic period (January 2018 through February 2020), adjusting for linear time trend and seasonality using weekly fixed effects. Counts were estimated overall and separately for each racial/ethnic group. Change in kidney failure incidence was defined as the difference between the estimated and observed numbers of patients with incident kidney failure, and relative differences between estimated and observed counts.

© 2021 Nguyen KH et al. *JAMA Network Open.*

References

1. US Department of Health and Human Services. End Stage Renal Disease Medical Evidence Report Medicare Entitlement and/or Patient Registration: Form CMS-2728-U3. *Centers for Medicare and Medicaid Services* 2006.
2. University of Wisconsin School of Medicine Public Health. In:2018 Area Deprivation Index v3.0.
3. Kind AJH, Buckingham WR. Making Neighborhood-Disadvantage Metrics Accessible - The Neighborhood Atlas. *N Engl J Med.* 2018;378(26):2456-2458.
4. Facts. U. Coronavirus locations: COVID-19 map by county and state. 2020; https://usafacts.org/visualizations/coronavirus-covid-19-spread-map/. Accessed March 16, 2021.
5. US Census Bureau. Annual Resident Population Estimates, Estimated Components of Resident Population Change, and Rates of the Components of Resident Population Change for States and Counties. 2020; https://www.census.gov/data/tables/time-series/demo/popest/2010s-counties-total.html.

© 2021 Nguyen KH et al. *JAMA Network Open.*
